# Enhancing the Security of the BB84 Quantum Key Distribution Protocol against Detector-Blinding Attacks via the Use of an Active Quantum Entropy Source in the Receiving Station

**DOI:** 10.3390/e25111518

**Published:** 2023-11-06

**Authors:** Mario Stipčević

**Affiliations:** Photonics and Quantum Optics Research Unit, Center of Excellence for Advanced Materials and Sensing Devices, Ruđer Bošković Institute, Bijenička cesta 54, 10000 Zagreb, Croatia; mario.stipcevic@irb.hr

**Keywords:** detector-blinding attack, quantum hacking, quantum key distribution, quantum entropy source

## Abstract

True randomness is necessary for the security of any cryptographic protocol, including quantum key distribution (QKD). In QKD transceivers, randomness is supplied by one or more local, private entropy sources of quantum origin which can be either passive (e.g., a beam splitter) or active (e.g., an electronic quantum random number generator). In order to better understand the role of randomness in QKD, I revisit the well-known “detector blinding” attack on the BB84 QKD protocol, which utilizes strong light to achieve undetectable and complete recovery of the secret key. I present two findings. First, I show that the detector-blinding attack was in fact an attack on the receiver’s local entropy source. Second, based on this insight, I propose a modified receiver station and a statistical criterion which together enable the robust detection of any bright-light attack and thus restore security.

## 1. Introduction

Randomness is the key ingredient in the security of any cryptographic protocol, including quantum key distribution (QKD) protocols. To understand the role of randomness in the security of QKD, I herein study the so-called *detector-blinding attack* (DBA) [1,2,3,4,5,6,7,8,9], which injects strong light into the quantum channel and exploits the technological weaknesses/features of the single-photon detectors used within with the purpose of controlling the internal entropy source and thus breaching the security. This particularly successful attack strategy makes it possible to not only eavesdrop completely undetected but also to fully recover the secret key, thus defying the two most notable strengths of QKD: (1) the ability to detect eavesdropping, and (2) the security of the generated secret key, which is unconditional according to information theory. Various adaptations of the DBA have been mounted on a range of scientific and commercial bipartite QKD systems [1,2] rendering them completely unusable even though their manufacturers had claimed that they were provably and unbreakably secure for years before the blinding attack became publicly known.

Each instance of DBA is specifically tailored to the detector feature that is being exploited, for example, passive quenching [5], active quenching [4], superlinearity [5], and thermal effects [1,2,5], as well as the specific architecture of the receiving station, such as passive or semi-active base control. In the demonstrated attacks the optical power of the incoming blinding light ranges from up to 10 mW continuous light [7], 8 mW continuous pulsing [4], all the way down to a few pW continuous pulsing [5] or even less than 120 photons per pulse [6], which is a power span of over 15 decades! Most scientific and research works propose defending against a detector-blinding attack by upgrading hardware so as to be able to detect strong light incoming from the quantum channel [10,11,12,13,14]. Apart from being technologically challenging, the main problem with this approach is that while it may prevent a specific attack, it does not guarantee the restoration of provable security, as noted in [4] and experimentally demonstrated in [14]. Repelling the attack via “brute force”, i.e., without a deeper understanding of how and why it works, leaves a bitter aftertaste which suggests that a clever modification of the attack could make it work again.

In this paper, I investigate the information-theory-based background of this devastating attack strategy on so-called “discrete variable QKD”, that is, QKD protocols which communicate via single qubits. I show that the success of a blinding attack relies on the attacker’s ability to obtain information about the receiver’s choice of detection base. A detector-blinding attack is achieved by actively sending a combination of a blinding light and a multi-qubit message with the purpose of taking control of the internal entropy source that selects the base. Based on this insight, an elegant defense strategy is developed for preventing the whole family of multi-qubit attacks on a receiver’s entropy source, restoring the unconditional security of a QKD protocol. Additionally, by monitoring an additional parameter of the communication over the quantum channel, it is possible to detect a general entropy source attack at no further cost with respect to hardware.

Even though blinding attacks work well on virtually any QKD protocol [8], for the sake of simplicity and without the loss of generality, I will study attacks on two implementations of the BB84 protocol [15,16] which were broken in [4,5].

## 2. The BB84 Protocol in a Nutshell

**Figure 1:**
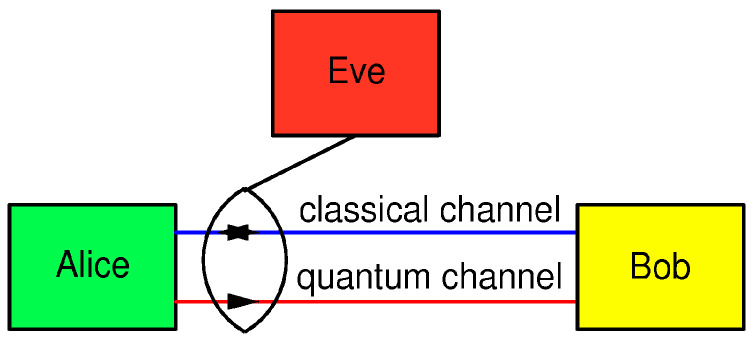
The BB84 protocol scenario. Alice, the sending station, generates qubits and sends them to Bob through the one-way quantum channel (originally, it was a 30 cm long air gap). Bob, the receiving station, receives and measures the qubits. To accomplish the generation of a secret key, an authenticated, but not necessarily secret, two-way classical communication channel between them is necessary. It is assumed that an eavesdropper, Eve, has a physical access to both channels and any equipment allowed by the laws of nature.

In the BB84 QKD protocol, two legitimate parties who wish to establish a secure communication, usually named Alice and Bob, are linked via one quantum channel and one authenticated (but not necessarily encrypted) classical channel, while an eavesdropper, Eve, has a physical access to both channels, as shown in Figure 1.

It is assumed that the classical communication channel is bidirectional and authenticated via an information-theoretic approach; Alice can be sure that she is talking to Bob and vice versa. The messages exchanged between Alice and Bob over this channel need not be encrypted, which means that Eve can read them but cannot change them. The quantum channel is unidirectional; Alice uses it to send qubits to Bob. 

In the first phase of the BB84 protocol, using the quantum channel, Alice sends Bob a random, classical bit equiprobably valued 0 or 1 and encoded in a linearly polarized qubit as follows: a bit with a value of 0 is randomly and equiprobably encoded with a polarization of either 0° or 45°, while a bit with a value of 1 is randomly and equiprobably encoded with a polarization of either 90° or 135°. Alice typically uses an electronic quantum random number generator (QRNG) [17,18,19,20,21,22] in order to generate a qubit. Bob randomly selects one of the two orthogonal bases in which the qubit is measured: (0°, 90°) or (45°, 135°). With a probability of 1/2, namely, when his base happens to match the polarization of the qubit, Bob obtains the exact bit value that Alice sent, while in the other case, Bob obtains a random bit. To figure out which is the case, Bob discloses his base to Alice over the authenticated classical channel. Knowing which qubit she sent, Alice replies either “keep”, in which case they keep the bit, or “discard”, in which case they both discard their bits. This communication is repeated until Alice and Bob collect a sufficiently long raw key, for example, until the length of L bits, agreed upon beforehand, is reached. The repeated communication of qubits is performed periodically with a frequency $f_{G}$, which technically allows Bob to define a short period of time $t_{G}$ (henceforth referred to as the *gate time*) around the expected time of arrival of the qubits and, in this manner, reduce the effect of noise (dark counts). Note that by listening to the communication over the classical channel alone, Eve is not able to figure out the value of the bit that is kept. Furthermore, she cannot fake any of the communication over the classical channel because it is authenticated. All she can do is to intervene in the quantum channel and listen to the classical channel. Ideally, after this initial phase, Alice and Bob have the same stream of bits, namely, their secret common key. However, due to inevitable hardware imperfections such as noise (dark counts and afterpulses in single-photon detectors), losses, and base misalignments, as well as Eve’s tampering with the quantum channel, there will be some differences (errors). In order to correct them, they need two further classical communication phases, namely, information reconciliation and privacy amplification, which are performed over the authenticated classical channel.

A blinding attack is performed entirely in the initial phase in which the quantum channel is used, so I do not need to analyze the two leftover classical phases of the BB84 protocol. Furthermore, in a DBA, Alice is never under attack, so her setup will not be discussed.

## 3. A Detector-Blinding Attack on the BB84 with Passive Base Selection

**Figure 2:**
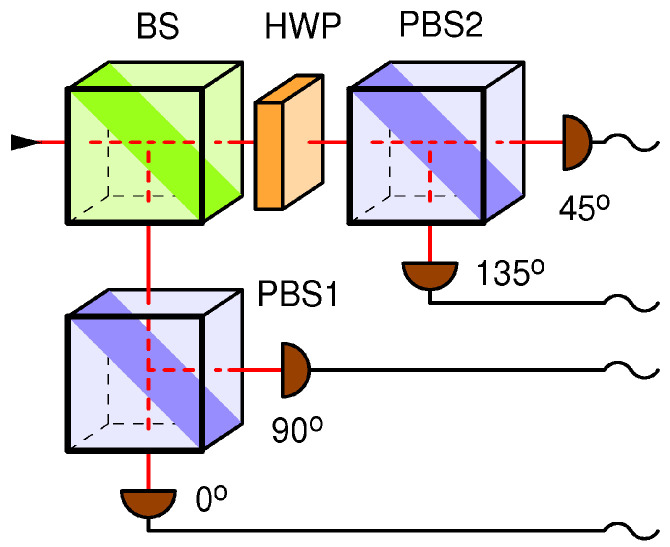
A receiver for the BB84 protocol with a passive random number generator; the measurement basis is selected randomly via the first polarization-insensitive beam splitter (BS). Each base consists of a polarizing beam splitter (PBS) and two detectors. The (45°, 135°) base is realized by placing a properly rotated half-wave plate (HWP) in front of it.

In the receiving station (Bob), which was broken in Ref. [4], the random selection of bases is performed passively with the help of a non-polarizing beam splitter (BS), as shown in Figure 2. Note that the sender (Alice) needs an equivalent of two random bits to generate the qubit state (namely, to choose one out of four possible qubit states), while Bob needs an equivalent of one bit of entropy per received qubit to randomly choose its measurement basis (one out of two possible bases), which mandates that each station must have a private random number generator or something equivalent. In the Bob setup, show, in Figure 2, the random selection of the receiving base is achieved through the use of the beam splitter (BS). 

A detector blinding attack (DBA) on this setup works as follows. Eve cuts the quantum channel between Alice and Bob (e.g., an optic fiber or aerial link) and simultaneously blinds all four detectors in Bob by shining a strong, pulsed, circularly polarized light of a high enough intensity, as explained in Ref. [4]. Each detector in Bob receives one-quarter of the incident power of the blinding light. The pulses are powerful and frequent enough (~70 kHz repetition rate; 8 mW during a pulse) to keep the detectors thermally blinded at all times during the qubit exchange. According to Ref. [4], this blinding strategy generates a four-fold coincidence among all detectors upon each blinding pulse.

In a blind state, detectors are sensitive only to strong pulses of light brighter than some threshold power $\;P_{thr}$ superimposed on top of the blinding light: so-called “fake states”. For example, Eve can shoot a pulse polarized at 45° and of a power just over ${2P}_{thr}$. Half of the power would end up in the 45° detector, making it click. The 135° detector will receive negligible power while the detectors in the other base will receive slightly more than $\frac{1}{2}P_{thr}$ each; thus, none of the other detectors will click. Note that the success of the attack is not very sensitive to the pulse power as it can be anywhere between just above ${2P}_{thr}$ and just below ${4P}_{thr}$, leaving quite a wide range for the attack to work on all four detectors which, for other security reasons, should have well-matched specifications anyway.

Eve measures Alice’s qubit in a randomly selected base (either (0°, 90°) or (45°, 135°)), using a receiving station which is a close copy of Bob. Note that Eve receives Alice’s qubits using the close copy of Bob and using the same protocol as Bob. After the measurement, Eve sends Bob a fake state matching her measurement, effectively copying her measurement result to Bob, who then measures the same as Alice. 

Finally, Eve passively listens to the classical channel between Alice and Bob and copies their activities the next two classical phases, namely, information reconciliation and the privacy amplification, in order to arrive to exactly the same “secret” key. Note that the attack does not introduce any extra bit error rate (BER). Since blinded detectors do not produce dark counts, a prudent Eve may send Bob random fake-noise pulses targeting each detector separately (at individual dark count rates), thus not taking chances in case Bob is monitoring the dark count rates of the detectors. Note that by Kerchoff’s principle of cryptography [23], Eve knows the dark count rates of Bob’s detectors. She could, for example, in quite a realistic scenario, have been the vendor of Bob’s station and could have measured the dark count rates (and all other relevant parameters) prior to selling it.

While it was deemed in Ref. [4] that detector blindability is what makes the DBA viable, in this work, I go a step further and seek insight at the level of the information theory. 

First, I note that in order for the DBA to work, Eve must make sure that her and Bob’s detection bases match *before* sending him a light signal that contains information. In the attack described above, the fake states contain the information Eve wants Bob to receive, while the continuous, circularly polarized blinding light does not contain any.

Next, I note that a fake state pulse is split deterministically at the beam splitter (BS) such that exactly one-half of its power hits each detection base. This is in stark contrast with the BB84 in which Bob receives a single qubit from Alice and the BS serves as an internal and private entropy source which randomly selects one of Bob’s two detection bases. Apparently, due to the deterministic splitting of a fake state under the DBA, the entropy of this internal randomness generator is zero—the entropy source is effectively disabled. But, without the random selection of bases, Bob is unable to run the BB84 protocol. Whatever Bob is actually running is *not* the BB84, but he does not know this because he does not realize his entropy source has been compromised.

In the BB84 protocol, the random base selection of the receiving station is a pivotal and indispensable facet of security. Let us investigate this in more detail. As explained above, as per the original BB84, Bob must calculate his reception base in order to be able send this information to Alice. He performs this based on his detections. Namely, if either the 0° or 90° detector fires, Bob concludes that his random base is 0; if any the other two detectors fire, then the base is 1; and finally, if no detector fires or more than two detectors fire (e.g., due to noise), then the communication instance is inconclusive and must be discarded. If Bob is monitoring the randomness of the sequence of bases (although this is not a part of the BB84 protocol), he would notice perfect randomness, but how could this occur when his own entropy source is incapacitated? Obviously, the randomness of his detection bases can be traced back to the beam splitter BS in the Eve station, which is still functioning in the quantum regime because it is receiving single qubits from Alice. This means that Eve and Bob are now logically united as one receiving station, “Eve-Bob”, which carries out a correct BB84 protocol with Alice. In effect, Eve has sneaked into Bob (even though she might be physically far away) and is able to obtain enough information to arrive at the exact same key as Alice and Bob.

What information does Eve obtain? The DBA allows Eve to directly set the detection outcome of any of the four detectors in Bob; thus, she knows everything that Bob knows. Indirectly, she also gains knowledge of Bob’s bases. It is tempting to conjecture that if Bob is monitoring the proper operation of his detectors, e.g., by making sure that they are not blinded and are sensitive to single photons, then devastating attacks like a DBA are impossible. 

However, could Eve mount a successful attack without blinding the detectors? To answer this question, I consider two scenarios. 

In the first scenario, let us suppose that Eve is able to set Bob’s receiving base via some means other than detector blinding. Then, her best strategy would be to set Bob’s base to match hers, but this time, she would send Bob a single qubit matching her measurement. Bob would detect the same bit as Eve without any increased loss or error with respect to when Eve is absent. Thus, Eve would obtain all the information she would obtain via the DBA and remain invisible. 

In the second scenario, Eve succeeds without having control over Bob’s bases. Let us suppose that Bob himself is choosing his reception base randomly (e.g., with the help of a true random number generator or its equivalent) but that Eve somehow obtains information about the base before receiving Alice’s qubit. Now, Eve may set *her* base to match Bob’s, receive Alice’s qubit, and send Bob a qubit that matches her measurement. Eve would again obtain the key and remain invisible. Note that in both of those scenarios, communication is performed via single qubits, and thus Bob’s BS will operate in the quantum regime and will introduce a 3 dB loss of useful signal. However, this amount of loss is not a problem for Eve; this will be clarified later.

From this discussion, one can see that if Eve obtains the information about Bob’s bases on time, the whole QKD protocol is doomed—it would provide no security whatsoever with or without the blinding of the detectors. I conclude that Eve’s success with the DBA relies solely on the fact that her mutual information with respect to Bob’s choice of bases is maximal:

(1) $$I\left(E,\;B\right)=1$$

where $E=\left(e_{1},{\ldots e}_{L}\right)$ is a random variable describing Eve’s knowledge about Bob’s receiving bases during all L rounds of the initial phase of the BB84 protocol, and $B=\left(b_{1},{\ldots b}_{L}\right)\;$ is a random variable describing Bob’s knowledge about his receiving bases. The base codes $e_{i}$ and $b_{i}$ have a value of 0 for the (0, 90) base and a value of 1 for the (45, 135) base.

The DBA is simply a technique through which Eve obtains the mutual information of Equation (1) in full.

## 4. A Detector-Blinding Attack on the BB84 with Semi-Active Base Selection

**Figure 3:**
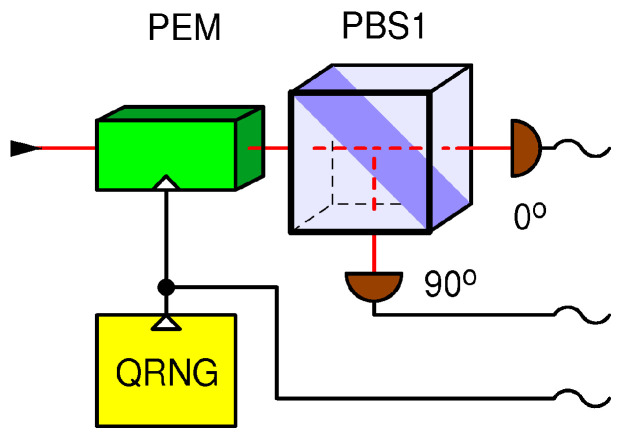
A receiver for the BB84 protocol with an active receiver with a phase electro-modulator (PEM); the measurement basis is determined by the quantum random number generator (QRNG) controlling the PEM.

In the receiving station (Bob), which was broken in Ref. [5], the random choice of detection bases is performed actively by means of a phase electro-modulator (PEM) driven by an explicit electronic QRNG, as shown in Figure 3. The state of the QRNG (0 or 1) determines the receiving base. According to the above discussion, I assume that the QRNG is private, meaning that it cannot be manipulated or predicted by Eve. This base choice technique functions exactly as required by the BB84 protocol for single qubits. It also diverts all the incoming power of a strong fake state to the selected base.

The version of DBA used against this setup works as follows. Eve again cuts the quantum channel between Alice and Bob. She simultaneously blinds all four detectors in Bob by shining a strong, continuous (CW), circularly polarized light of a carefully tailored intensity, as explained in Ref. [5]. Note that the circularly polarized light distributes evenly among the detectors regardless of the state of the PEM, and Eve is thus able to simultaneously blind and keep blinded forever (or at least during the qubit exchange phase) both detectors and thus both bases. Note that while the active control of the PEM works correctly for qubits and fake states, it has no effect on the blinding light. Therefore, I refer to the setup in Figure 3 as “semi-active”. As in the previous DBA, the blinded detectors are sensitive only to fake state pulses brighter than a threshold power $P_{thr}$ superimposed on top of the blinding CW light, which confines the fake state power to the range between slightly above ${2P}_{thr}$ and slightly below ${4P}_{thr}$.

The difference from the previous setup (shown in Figure 2) is that here, Eve is able to copy her measurement to Bob with only 50% success, namely, in those instances in which her base coincides with Bob’s by chance. In the other 50% of instances, Bob’s blinded detectors receive nothing, and the bit is lost. Next, by passively listening to the classical communication between Alice and Bob in the next phases of the BB84 protocol, Eve is able to figure out which bits were lost, sift the same bits as Alice and Bob, and recover 100% of the key. While this attack reduces the key rate by a factor of two (equivalent to a channel loss of 3 dB) with respect to when Eve is not there, the security situation is not satisfactory because Eve still obtains the full key and, in the presence of other losses or a strongly varying loss, she might remain undetected. In fact, Eve might place herself away from Bob such that the channel loss between them is at least 3 dB, in which case the loss would be compensated for because Bob detects her fake states with no loss. In fact, if the channel loss between Eve and Bob is greater than 3 dB, Eve will need to fake an additional loss so as not to raise suspicion, which is technically quite easy. Additionally, note that Alice and Bob have no secure means of calibrating the key rate because Eve might decide to be present at all t times, introduce the 3 dB loss when not eavesdropping, and remove it when she does. This receiving station (Figure 3) architecture was also shown to be vulnerable to the weak pulse (~120 photons) attack via an early test prototype using superconducting nanowire single-photon detectors, exploiting their superlinear behavior and/or their great sensitivity to thermal effects [6].

The technical reason for this relative success of Eve even with the “semi active” control of the bases is that regardless of the state of the PEM, the incident circularly polarized light is equally distributed among the four detectors, which enables Eve to keep them blinded all at all times. The theoretical problem is that it does not satisfy assumptions under which the BB84 protocol has been proven secure (e.g., [16,24]), namely, that light received by Bob’s station must hit *no more than one* base at a time, which is an implicit assumption of single-qubit communication. This assumption is violated in the setup in Figure 3.

## 5. Improved Setup with a Fully Active Base Selection

**Figure 4:**
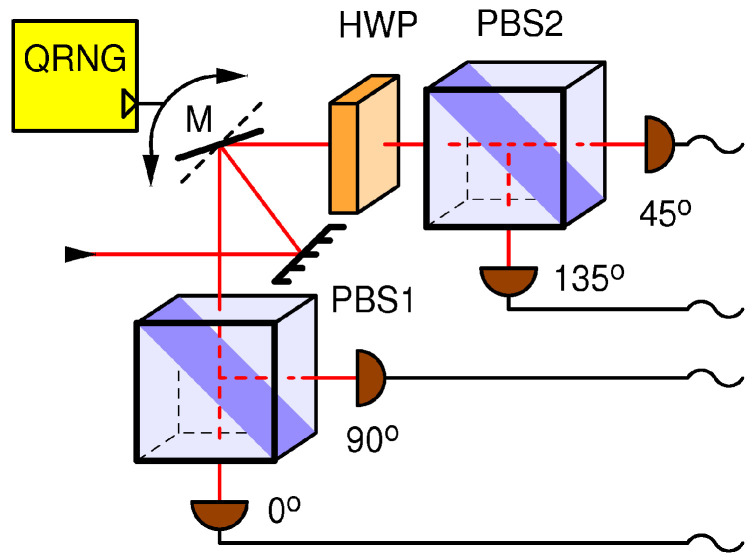
Receiver for a BB84 protocol with an exclusive active receiver; the receiving basis is determined by the quantum random number generator QRNG, which flips the mirror (M) between two possible angles, each of which reflects *all* of the light into one and only one measurement basis.

Following the discussion above, I propose an improved receiving station, shown in Figure 4. It consists of two physically distinct detection bases and a mirror which directs *all* incoming light to *one and only one* base under the control of a local and private QRNG.

This switching mechanism is allowed by the laws of quantum mechanics and could be implemented, for example, via the use of a motorized mirror, a MEMS router [25,26], a mechanical switch inside optical fibers [27], or an electro-optically controlled Mach–Zehnder interferometer (MZI) 2 × 2 port switch [28]. While micromechanical mirror switches offer switching times as low as a microsecond, an MZI offers sub-nanosecond switching times and is suitable for photonic integration. Whichever technique is used, it is imperative that it is insensitive to polarization and does not introduce high losses. For all of these techniques, insertion losses of a fraction of a dB have been demonstrated, which is quite acceptable given the total loss budget of a typical quantum channel. For simplicity without a loss of generality, I assume that the four detectors operate in a free-running mode, meaning that they are ready to detect at all times except during the dead time. In particular, this means that gating is assumed to be implemented at the level of data analysis, not at the level of disabling the optical sensors. If the latter is preferred, for example, to reduce noise, a modification using double-gating can be implemented that would preserve sensitivity to out-of-gate thermal blinding. I will now analyze how this setup operates under a DBA attack.

A DBA attack requires the careful tailoring of the characteristics of the blinding light (e.g., its power, polarization, and whether it should be a CW or a pulsed light) as well as the precise modeling of single-photon detectors in order to arrive at a reliable blinding technique for a specific receiving station. The novelty of the proposed receiver (shown in Figure 4) with respect to the two previous receivers (shown in Figure 2 and Figure 3) is that here, only one (randomly selected) base receives light, while the other base is in darkness. Following the previous discussion, I will differentiate two cases: continuous blinding light and pulsed blinding light. 

In the case of a **continuous blinding light** attack on the new receiver, Eve cannot keep both bases blinded at all times, nor is her attack invisible. Namely, whenever the QRNG changes the receiving base, the two detectors that were previously in the dark are both simultaneously hit by a strong blinding light. One is able to predict what happens after without modeling any a particular detector. When a single-photon sensitive detector which was previously in the dark and ready to detect is hit by a strong light, it will detect, that is, produce a “click”, an output pulse signifying a detection, at least once. This is because the sensor will perform exactly its intended function when a single photon is detected; for example, an avalanche photodiode will become highly conductive, while a superconductive nanowire will become highly resistant. These transitions will necessarily generate a detection signal. The detector(s) in question may become blinded, but that will only happen *after* the initial detection. As explained before, due to an inability to decide which state was sent, Bob will have to discard such an event, and this will happen in 50% of gates. For the other 50% of gates, Eve is able to send her measurement to Bob with a probability of one-half (in total, 25% of gates), namely, in those instances in which her base coincides with Bob’s randomly selected base by a chance, while for the remaining 25% of gates, Bob will receive nothing (his detectors will remain silent), resulting in a four-fold drop in the effective key rate with respect to no eavesdropper. Even though this higher loss introduced by Eve, equivalent to 6 dB, is, in principle, easier to for Alice and Bob to detect, the situation is still unsatisfactory because (a) Eve may be able to compensate for the loss (as explained before), and (b) Eve can obtain complete knowledge of the key without being detected.

In the case of a **pulsed blinding light**, very strong laser pulses keep the detectors thermally blinded. In the original DBA described in Refs. [4,7], which was performed on the setup shown in Figure 2, it was noted that such strong pulses will cause simultaneous detections in all affected detectors. For this reason, Eve keeps the blinding pulses outside of gates so they do not generate extra in-gate coincidences and sends them frequently enough to keep all the detectors blinded at all times. However, when this attack is applied to the setup in Figure 4, Eve is not sure which base is blinded because the bases are selected at random with a probability of one-half; therefore, she needs to increase the blinding rate by at least twice. Even so, because of the binomial statistics of the base selection, a base might occasionally escape blinding and generate an extra in-gate coincidence when hit by a fake state. While the details of this behavior need to be investigated further, it is safe to assume that in the best scenario for Eve, she will be able to achieve blinding and retrieve the full key, albeit at the expense of a 6 dB loss caused merely by the random base choice. The unavoidable price paid for the pulsed blinding causes out-of-gate coincidences between the two detectors in the active (selected) base.

### 5.1. Attack Analysis

From the discussion above, it would naïvely seem that Eve can successfully eavesdrop on the receiver in Figure 4 using blinding techniques. However, this particular setup enables yet another option available to Alice and Bob for defeating Eve which relies on monitoring in-gate and out-of-gate coincidences. 

Let us first discuss a regular quantum communication using single qubits in which Bob knows the arrival time of Alice’s qubit and opens a short detection time window $t_{G}$, called the “gate”, around it to detect the qubit. In order for the BB84 protocol to have a non-zero secret key rate, the bit error rate of the first phase must be <11.5% [29]. The dark count occurrence probability during the gate for a single detector is

(2) $$p_{DCR}={\left(1-e^{-t_{G}f_{DCR}}\right)≅t}_{G}f_{DCR}$$

where $f_{DCR}$ is the dark count rate (DCR) of the detector and $t_{G}$ is the duration of the gate. The right-hand approximation is valid for $t_{G}f_{DCR}\ll 1$, which is often the case. Bob can confidently determine the dark count rates for all four detectors by disconnecting the receiver from the quantum channel and performing measurements in the dark. To simplify the calculation, I will henceforth assume equal DCR values for all detectors. A DCR-caused “accidental” coincident detection between a pair of detectors belonging to the same base is a Poissonian random event. Its probability during the gate-open period $t_{G}$, or its “in gate” probability, is given by

(3) $$p_{AIn}=p_{DCR}^{2}≅t_{G}^{2}f_{DCR}^{2}$$

When receiving qubits, the coincidence rate between two detectors forming a base is described by the Hong–Ou–Mandel (HOM) effect, which characterizes the probability of simultaneous photon detection at the two outputs of a beam splitter [30]. By considering a coherent input state qubit emitted from Alice with an average photon number $\langle n\rangle$ within a gate-open period $t_{G}$, the per-gate coincidence probability measured by Bob in any of its two bases is given by [31]:

(4) $$p_{QIn}=\frac{1}{2}{\left(1-e^{-\frac{\langle n\rangle \epsilon_{B}\epsilon_{QCh}}{2}}\right)}^{2}≅\frac{{\langle n\rangle }^{2}\epsilon_{B}^{2}\epsilon_{QCh}^{2}}{8}$$

where $\epsilon_{B}$ is Bob’s system quantum efficiency, which includes the effects of the finite detector’s quantum efficiency, optics imperfections, and other losses in Bob, while the quantum channel efficiency $\epsilon_{QCh}$ is a fraction of the qubits that survive through the quantum channel connecting Alice and Bob. The right-hand approximation is valid for $\left({\langle n\rangle \epsilon }_{B}\epsilon_{QCh}\right)/2\ll 1$, which holds true in a practical QKD in which typically $\langle n\rangle \;\sim \;0.1$ and $\epsilon_{S\;}\sim \;0.1-0.5,$ while losses can be anywhere between a few dB and several tens of dB. The overall factor 1/2 equals the probability that the qubit is in a “wrong” base. In the case in which the base is “right” for the given qubit, there will be no coincidence in principle because the photon will end up in only one detector. Here, I neglect a possible imperfection in Bob’s polarizers which in may allow for a small probability of coincidence (typically less than 0.01). The coincidence probability is composed of both the accidental and qubit-related coincidences. As both are Poisson random processes, the total in-gate coincidence probability $p_{Cin}$ for the regular BB84 protocol in setup in Figure 4 is given by

(5) $$p_{CIn}=p_{AIn}+p_{QIn}-{p_{AIn}p}_{QIn}≅\;t_{G}^{2}f_{DCR}^{2}+\frac{{\langle n\rangle }^{2}\epsilon_{B}^{2}\epsilon_{QCh}^{2}}{8}$$

As an example, assuming the following realistic values of a gate time $t_{G}=5$ ns, a dark count rate $f_{DCR}=1000$ cps, an average photon number in a qubit $\langle n\rangle =0.1$, Bob’s system quantum efficiency $\epsilon_{B}=0.25$, and a quantum channel transfer efficiency $\epsilon_{QCh}=0.16$ (−8 dB), one arrives at $p_{CIn}={2\times 10}^{-6}$.

Using the same approach, I can calculate the out-of-gate coincidence rate for the DCR:

(6) $$p_{AOut}={\left(\frac{1}{f_{G}}-t_{G}\right)}^{2}f_{DCR}^{2}.\;$$

The qubits do not fall outside of a gate and therefore do not cause out-of-gate coincidences.

(7) $$p_{QOut}=0.$$

The total out-of-gate coincidence probability for the regular BB84 protocol in setup in Figure 4 equals

(8) $$p_{COut}=p_{AOut}+p_{QOut}-p_{AOut}p_{QOut}={\left(\;\frac{1}{f_{G}}-t_{G}\right)}^{2}f_{DCR}^{2}$$

where $f_{G}$ is rate at which Alice emits qubits. Using the same set of communication parameters as in the previous example and $f_{G}=10$ MHz, one arrives at $p_{COut}=9.0\times 10^{-9}$. 

Our defense strategy relies on the fact that both the in-gate and out-of-gate coincidence probabilities, described in Equations (5) and (8), are dramatically enhanced under a strong-light attack, as will be shown below. 

**The case of a CW DBA.** Let N be the number of photons arriving during a gate onto a detector. Then, the probability $p_{N}$ that the detector with a quantum efficiency ϵ will detect at least once is given by [31]

(9) $$p_{N}=1-{\left(1-\epsilon \right)}^{N}$$

or even closer to unity in the case of a superlinear detector [6]. Considering a coherent blinding light with an average photon number 〈m〉 arriving on Bob during a gate, the overall coincidence probability, according to Equation (9), is

(10) $$p_{DBAIn}={\left(1-{\left(1-\epsilon_{B}\right)}^{\frac{\langle m\rangle }{2}}\right)}^{2}.$$

Assuming an attack using as few as $\langle m\rangle =20$ photons incident on Bob per gate, and assuming that $\epsilon_{B}=0.25$, then the coincidence probability $p_{DBAIn}$ is about 0.94, and it would quickly approach a theoretical maximum of 1 for a larger $\langle m\rangle$. This is about six orders of magnitude higher than the in-gate coincidence probability of the regular BB84 protocol, $p_{CIn}={2\times 10}^{-6}$, as calculated above, even though the incident optical power considered in this example is much smaller what is required for an actual detector-blinding attack.

**In the case of a pulsed DBA**, which operates on the principle of thermal blinding with strong out-of-gate pulses, such an attack will not enlarge in-gate coincidences between detectors in the same base. However, as explained above, each blinding light pulse will create one out-of-gate coincident pair of detections in two detectors belonging to the base which is currently selected by the QRNG. The coincidence probability for either detection base, per gate, is given by

(11) $$p_{DBAOut}=\frac{f_{BPR}}{f_{G}}$$

where $f_{BPR}$ is the minimum required blinding pulse rate, 70 kHz according to [4], and, as explained above, this must be at least doubled by Eve because of the active base selection in this setup. As justified above, I do not add dark counts to that rate. 

Assuming $f_{BPR}=140$ kHz and that the other parameters are the same as in the previous examples, I obtain $p_{DBAOut}=1.4\times 10^{-2}$. On the other hand, the coincidence probability of the undisturbed QKD $p_{COut}=9.0\times 10^{-9}$, as calculated above, is more than six orders of magnitude smaller.

This demonstrates that for both in-gate and out-of-gate DBAs, the corresponding coincidence probabilities will be strongly enhanced with respect to the undisturbed BB84 when using the setup shown in Figure 4. This fact is the basis for my defense strategy, which should work for any type of strong-light attack, including a DBA.

The high ratio of coincidence probabilities under the DBA and in a normal communication is robust across a realistic range of communication parameters. For example, in an extreme state-of-the-art communication with a qubit rate $f_{G}=1$ GHz and a gate time $t_{G}=200$ ps, the in-gate ratio stays the same while the out-of-gate ratio becomes 100 times greater. In fact, if all other parameters remain constant, the in-gate ratio is almost independent of $f_{G}$ and $t_{G}$, while the out-of-gate ratio is roughly proportional to $f_{G}$ in the range between 1 and 1000 MHz.

### 5.2. Proposed Defense Strategy against Strong-Light Attacks

**Figure 5:**
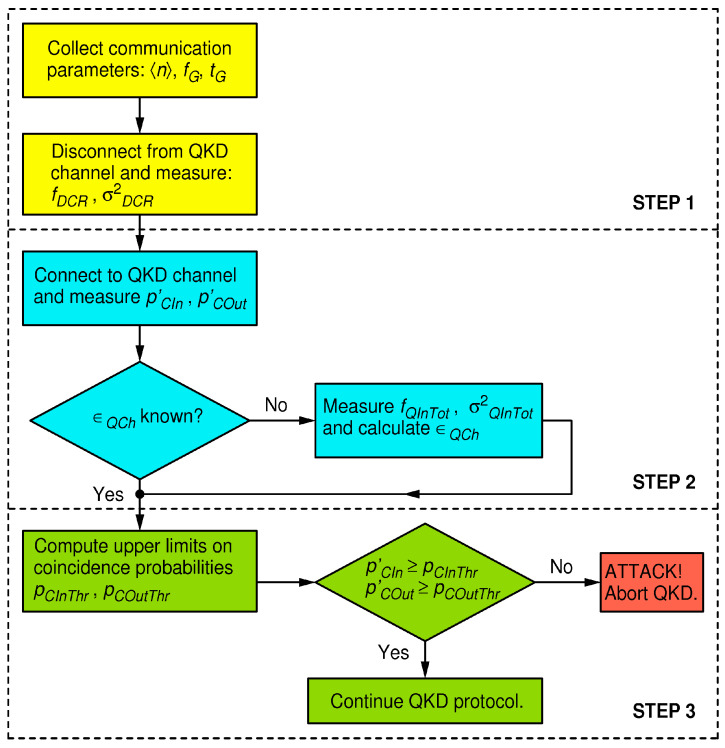
Flow chart of the defense strategy for the proposed receiver in Figure 4.

To defend against strong-light attacks, I focus on two pairs of equations: Equations (5) and (8), which model the in- and out- of-gate coincidence probabilities during the execution of a pristine BB84 QKD protocol, and Equations (10) and (11), which model the same coincidence probabilities but under a strong-light attack. The huge increase in the coincidence probabilities under a strong-light attack, even for an injected light level far less than what is required to mount an actual blinding attack, presents a robust figure of merit for discerning the undisturbed quantum communication from an attack condition. In modern QKD systems, the output of each detector is connected to a dedicated channel of a time-tagger, and time stamps are recorded; thus, the above probabilities can be evaluated using a simple data analysis. The defense strategy, which is valid for the receiver in Figure 4, is outlined by the flow chart in Figure 5, and the details are described below. 

**In the first step**, one collects information about Bob’s system quantum efficiency $\epsilon_{B}$, the dark count rates $f_{DCR}^{\left(k\right)}$ of all four detectors, where the index $k\in \left\{0,1,2,3\right\}$ marks the detector dedicated to measuring the k-th polarization (namely, the linear polarization of an angle $\frac{\pi }{4}k$), and estimates their respective statistical uncertainties $\sigma_{DCR}^{\left(k\right)}$. As before, in order to simplify treatment but with an obvious extension to the general case, I henceforth assume that all detectors exhibit the same dark count parameters:$\;f_{DCR}$ and $\sigma_{DCR}$. All these parameters may be known in advance. Alternatively, one may disconnect Bob from the quantum channel and perform an appropriate set of measurements (calibration) to obtain them. I also assume that the parameters $\langle n\rangle$, $f_{G}$, $t_{G}$ are known or negotiated before the start of the QKD protocol. While Eve may know all of these parameters (by Kerchoff’s principle), she may not influence them. 

**In the second step**, during the quantum communication session (namely, the first phase of the BB84), one measures Bob’s in-gate and out-of-gate coincidence probabilities related to the reception of qubits, namely, $p_{CIn}^{\prime }$ and $p_{COut}^{\prime }$. For example, $p_{CIn}^{\prime }$ is evaluated as the sum of rates of coincidences between pairs of detectors (0, 2) and (1, 3) that happen only during gates divided by the rate of gates $f_{G}$. The evaluation of $p_{COut}^{\prime }$ is similar except that the coincidences are now counted out of gates. Furthermore, if the quantum channel transmissivity $\epsilon_{QCh}$ is not known/calibrated beforehand, it should be estimated. To that end, one measures the total rate of detections for all four detectors within a coincidence window $f_{QInTot}$ and estimates its variance $\sigma_{QInTot}^{2}$. The qubit reception probability is then equal to

(12) $$p_{QInTot}=\frac{f_{QInTot}-f_{G}t_{G}\sum_{k=0}^{3}f_{DCR}^{\left(k\right)}}{f_{G}}=\frac{f_{QInTot}}{f_{G}}-4t_{G}f_{DCR}$$

According to Equation (10), for an undisturbed BB84, it should be equal to

(13) $$p_{QInTot}=1-{\left(1-\epsilon_{B}\epsilon_{QCh}\right)}^{\langle n\rangle }.$$

Solving for $\epsilon_{QCh}$ gives

(14) $$\epsilon_{QCh}=\frac{1}{\epsilon_{B}}\left(1-{\left(1-p_{QInTot}\right)}^{\frac{1}{\langle n\rangle }}\right).\;$$

Note that Eve does not control the variables $\epsilon_{B}$ or $\langle n\rangle$, but she can manipulate the apparent $p_{QInTot}$ to a certain extent. On one hand, she could lower $p_{QInTot}$ by randomly omitting qubits from Alice, but that would not enable her to increase her information about the resulting key, while the key would only become shorter for all three parties. In addition, the communication-induced coincidence probabilities would become smaller, and thus the ratio of the probability of blinding-induced coincidences would increase in comparison to ones from the communication. This would make Eve’s attack more easily discoverable. On the other hand, she could increase $p_{QInTot}$ by removing a loss she induced beforehand or part of it, as explained previously, if any. In that case, she must “invent” new qubits for Bob that would cause an increase in the BER, causing Alice and Bob to remove this extra information in the privacy amplification so that Eve will not gain any new knowledge about the key and nor would the effective key length change. In this way, her discoverability would decrease by the factor with which she enlarged $p_{QInTot}$, but this is not a significant gain, as will be shown below.

Before moving to the final step, one needs to estimate the statistical uncertainty of $\epsilon_{QCh}$. To that end, I assume that the predominant uncertainty of $p_{QInTot}$ comes from the statistical uncertainties of $f_{QInTot}$ and the dark count rates. I further assume that qubit detection and dark counts are Poissonian events, it is then straightforward to show, using the standard statistical theory of the propagation of uncertainty, that its variance is given by

(15) $$\sigma_{QCh}^{2}=\left(\frac{\sigma_{QInTot}^{2}}{f_{G}^{2}}+16t_{G}^{2}\sigma_{DCR}^{2}\right){\left(\frac{{\left(1-p_{QInTot}\right)}^{\frac{1}{\langle n\rangle }-1}}{\langle n\rangle \epsilon_{B}}\right)}^{2}.$$

At the end of this step, the following parameters pertaining to the hardware and the quantum communication are known: $\langle n\rangle$, $f_{G}$, $t_{G}$, $\epsilon_{B}$, $f_{DCR}$, $\sigma_{DCR}$, $p_{QIn}^{\prime }$, $p_{QOut}^{\prime }$, $\epsilon_{QCh}$, and $\sigma_{QCh}$. These are input into the final step. 

**In the third and final step**, one calculates the upper limits on the probabilities of the in- and out-of-gate coincidence probabilities for an undisturbed QKD communication ($p_{CInTHR}$ and $p_{COutTHR})$ and compares them to the directly measured probabilities of the in- and out-of-gate coincidences ($p_{QIn}^{\prime }$ and $p_{QOut}^{\prime }$) in order to detect whether the QKD system is under attack at a desired (arbitrary) level of confidence. 

The in- and out-of-gate undisturbed coincidence probabilities $p_{CIn}$ and $p_{COut}$ can be estimated using Equations (5) and (8), respectively, and the parameters are available from the previous step. Their respective variances are

(16) $$\sigma_{CIn}^{2}=4t_{G}^{4}f_{DCR}^{4}+\frac{{\langle n\rangle }^{4}\epsilon_{B}^{4}\epsilon_{QCh}^{4}}{16}{\left(\frac{\sigma_{QCh}}{\epsilon_{QCh}}\right)}^{2}$$

(17) $$\sigma_{COut}^{2}=4{\left(\;\frac{1}{f_{G}}-t_{G}\right)}^{4}f_{DCR}^{4}{\left(\frac{\sigma_{DCR}}{f_{DCR}}\right)}^{2}\;.$$

Next, without any assumption about the statistical distribution(s) governing their values, one can find the respective upper limits on these probabilities, using Chebyshev’s inequality [32]

(18) $$p\left(p_{CIn}\geq p_{CInTHR}\right)\leq \frac{\sigma_{CIn}^{2}}{p_{CInTHR}^{2}}.$$

Keeping in mind that all variables in the above equation are positive, an upper limit on $\epsilon_{QCh}^{\prime }$ is given by

(19) $$p_{CInTHR}\leq \frac{\sigma_{CIn}}{\sqrt{p\left(p_{CIn}\geq p_{CInTHR}\right)}}$$

which can be interpreted as

(20) $$p_{CInTHR}=\frac{\sigma_{CIn}}{\sqrt{{1-p}_{CONF}}}$$

where $p_{CONF}$ is an arbitrarily chosen probability (confidence level) that inequality $p_{CIn}<p_{CInTHR}$ holds. Equivalently, I obtain

(21) $$p_{COutTHR}=\frac{\sigma_{COut}}{\sqrt{1-p_{CONF}}}.$$

Finally, I define the following security criterion. If the inequalities

(22) $$p_{CIn}^{\prime }<p_{CInTHR}\;p_{COut}^{\prime }<p_{COutTHR}$$

hold, one is to conclude that the QKD session is not under a DBA with a confidentiality level of $p_{CONF}$. On the other hand, if any of the two inequalities fail, then the session is insecure and should be aborted. 

Let us now work out one numerical example. I assume the same realistic parameters as above for an easier comparison, namely, $\langle n\rangle =0.1$, $f_{G}=10$ MHz, $t_{G}=5$ ns, $\epsilon_{B}=0.25$, $and\;f_{DCR}=1$ kcps; additionally, $\sigma_{DCR}=100$ cps, total in-gate detection rate $f_{QInTot}=4\times 10^{4}$ cps, and $\sigma_{QInTot}=1$ kcps. From Equations (12), (14) and (15), I obtain $p_{QInTot}=3.98\times 10^{-3}$, $\epsilon_{QCh}=0.156,$ and $\sigma_{QCh}=3.86\times 10^{-3}$. Then, Equations (16) and (17) yield $\sigma_{CIn}=9.4\times 10^{-6}$ and $\sigma_{COut}=1.8\times 10^{-9}$. Finally, even requiring a very high confidence level of 99.9999% $\left(p_{CONF}=0.999999\right)$, Equations (20) and (21) provide rather small/strict threshold values for the coincidence probabilities: $p_{CInTHR}=9.4\times 10^{-3}$ and $p_{COutTHR}=$ $1.8\times 10^{-6}$. 

The criterion in Equation (22) tells us that as soon as $p_{CIn}^{\prime }\geq 9.4\times 10^{-3}$ or $p_{COut}^{\prime }\geq 1.8\times 10^{-6}$, one concludes, with 99.9999% confidence, that the session has been compromised. To appreciate the discerning capability of this defense, I recall that for the DBAs described in the previous section, using the same communication parameter values, I determined $p_{CIn}^{\prime }\approx 0.94$ and $p_{COut}^{\prime }\approx 1.4\times 10^{-2}$, which is several orders of magnitude higher than their respective thresholds $p_{CInTHR}$ and $p_{COutTHR}$. I conclude that it is very unlikely that any multi-photon attack strategy would pass the criterion Equation (22) in conjunction with the setup in Figure 4.

## 6. Discussion and Conclusions

In this work, I investigate the merit of internal entropy sources for the security of QKD. To that end, I study a particularly vicious “detector blinding attack” (DBA) which was successfully demonstrated on the most frequently used architectures of QKD receivers used for the BB84 protocol, as described in the literature. By virtue of a DBA, an attacker (Eve) is able to obtain a perfect copy of the key agreed upon between two legitimate parties (Alice and Bob) while remaining undetected. While most of the defenses described in the literature consist essentially of detecting a strong light incoming from the quantum channel, the authors of the DBA have shown that such countermeasures can be defeated, as discussed in the introduction. Therefore, I took a different path. Noting that the necessary condition for the success of a DBA is Eve’s ability to gain control over the internal entropy source of the receiver station (Bob), I modified the setup such that the mentioned condition cannot be met. 

We show that even though the successfully attacked passive (Figure 2) and semi-active (Figure 3) receiver configurations, as well as the fully active (Figure 4) configuration proposed herein, are all seemingly functionally equivalent, only the latter is secure against attacks on the internal entropy source. Namely, a circularly polarized light incident on the passive or semi-active setup will distribute its power evenly between all detectors present in the setup. This gives Eve an opportunity to blind the detectors and consequently manipulate their detections. On the other hand, in the proposed fully active receiver, light never falls on all detectors simultaneously; it can only fall upon one detection base at a time. Whenever the blinding light is switched from one base to another which was previously in the dark, the two detectors belonging to that base will generate a coincident detection with a very high probability which is orders of magnitude higher than in the normal (undisturbed) quantum communication, in which correlations of that sort are highly suppressed by design. In essence, my attack defense consists of comparing the measured coincidence probability to the level expected from the undisturbed quantum communication; if it is higher, then the system is under attack.

In the passive and the semi-active setups, an information-theoretically perfect random number generator (QRNG), which is required for selecting the receiving base, is realized via a front-end non-polarizing beam splitter which can be directly accessed by Eve via the quantum channel. A subtle difference of my proposed setup is that it makes use of an explicit QRNG which collapses the wave function within itself and outputs entropy in form of classical information. Such an entropy source does not accept any information from the communication channels and consequently cannot be controlled nor predicted by Eve. Thus, I deter the DBA, and in fact any strong-light attack, at the conceptual level by disabling for Eve the possibility of manipulating or reading out the internal entropy source of Bob. 

The modified receiver setup and the statistical criterion for recognizing a strong-light attack are the main contributions of this work. From the rigorous mathematical treatment and examples, it is clear that the proposed statistical criterion has an overwhelming power to discern between a genuine quantum communication and an attack using a strong light. I believe that my approach of ensuring provable security, which is non-specific to any particular version of a DBA, should have greater resilience against modified DBAs that may be conceived in the future. 

Even so, I think that neither this, nor any other defense strategy, voids the necessity of monitoring the proper functioning of single-photon detectors, as well as all other active or passive components of both QKD stations, Alice and Bob, alike, and the characteristics of the quantum channel. All this is needed to keep the whole system compliant with security proof at all times. The absence of such a monitoring system, as well as the laconic use of an exposed beam splitter as an entropy source in the receiving station, creates a possibility for devastating attacks, as have been demonstrated on a number of scientific and commercial QKD systems. The lesson learned from this is that the real-world implementation of a QKD protocol should be carried out with the utmost care and constant monitoring of its compliance with a theoretical idealization.

## References

[1] Lydersen L., Wiechers C., Wittmann C., Elser D., Skaar J., Makarov V. (2010). Hacking commercial quantum cryptography systems by bright illumination. Nat. Photonics.

[2] Gerhardt I., Liu Q., Lamas-Linares A., Skaar J., Kurtsiefer C., Makarov V. (2011). Full-field implementation of a perfect eavesdropper on a quantum cryptography system. Nat. Commun..

[3] Lydersen L., Akhlaghi M.K., Hamed Majedi A., Skaar J., Makarov V. (2011). Controlling a superconducting nanowire single-photon detector using tailored bright illumination. New J. Phys..

[4] Sauge S., Lydersen L., Anisimov A., Skaar J., Makarov V. (2011). Controlling an actively-quenched single photon detector with bright light. Opt. Express.

[5] Makarov V. (2009). Controlling passively quenched single photon detectors by bright light. New J. Phys..

[6] Lydersen L., Jain N., Wittmann C., Marøy Ø., Skaar J., Marquardt C., Makarov V., Leuchs G. (2011). Superlinear threshold detectors in quantum cryptography. Phys. Rev. A.

[7] Lydersen L., Wiechers C., Wittmann C., Elser D., Skaar J., Makarov V. (2010). Thermal blinding of gated detectors in quantum cryptography. Opt. Express.

[8] Lydersen L., Skaar J., Makarov V. (2011). Tailored bright illumination attack on distributed-phase-reference protocols. J. Mod. Opt..

[9] Wiechers C., Lydersen L., Wittmann C., Elser D., Skaar J., Marquardt C., Makarov V., Leuchs G. (2011). After-gate attack on a quantum cryptosystem. New J. Phys..

[10] Yuan Z.L., Dynes J.F., Shields A.J. (2010). Avoiding the blinding attack in QKD. Nat. Photonics.

[11] Honjo T., Fujiwara M., Shimizu K., Tamaki K., Miki S., Yamashita T., Terai H., Wang Z., Sasaki M. (2013). Countermeasure against tailored bright illumination attack for DPS-QKD. Opt. Express.

[12] Si H., Liu H., Ma H. Optical Fiber Communication Network Eavesdropping and Defensive Measures. Proceedings of the 2nd International Forum on Management, Education and Information Technology Application (IFMEITA 2017).

[13] Wang J., Wang H., Qin X., Wei Z., Zhang Z. (2016). The countermeasures against the blinding attack in quantum key distribution. Eur. Phys. J. D.

[14] Acheva P., Zaitsev K., Zavodilenko V., Losev A., Huang A., Makarov V. (2023). Automated verification of countermeasure against detector-control attack in quantum key distribution. EPJ Quantum Technol..

[15] Bennett C.H., Brassard G. (1984). Quantum public key distribution system. Proceedings of the IEEE International Conference on Computers, Systems and Signal Processing.

[16] Bennett C.H., Bessette F., Brassard G., Salvail L., Smolin J. (1992). Experimental quantum cryptography. J. Cryptol..

[17] Jennewein T., Achleitner U., Weihs G., Weinfurter H., Zeilinger A. (2000). A Fast and Compact Quantum Random Number Generator. Rev. Sci. Instrum..

[18] Stefanov A., Gisin N., Guinnard O., Guinnard L., Zbinden H. (2000). Optical quantum random number generator. J. Mod. Opt..

[19] Stipčević M., Rogina B.M. (2007). Quantum random number generator based on photonic emission in semiconductors. Rev. Sci. Instrum..

[20] Wayne M.A., Jeffrey E.R., Akselrod G.M., Kwiat P.G. (2009). Photon arrival time quantum random number generation. J. Mod. Opt..

[21] Wahl M., Leifgen M., Berlin M., Röhlicke T., Rahn H.-J., Benson O. (2011). An ultrafast quantum random number generator with provably bounded output bias based on photon arrival time measurements. Appl. Phys. Lett..

[22] PKeshavarzian P., Ramu K., Tang D., Weill C., Gramuglia F., Tan S.S., Tng M., Lim L., Quek E., Mandich D. (2023). A 3.3-Gb/s SPAD-Based Quantum Random Number Generator. IEEE J. Solid-State Circuits.

[23] Kerckhoffs A. (1883). La Cryptographie Militaire. J. Sci. Mil..

[24] Shor P.W., Preskill J. (2000). Simple proof of security of the BB84 quantum key distribution protocol. Phys. Rev. Lett..

[25] Savastano L., Maier G., Pattavina A., Martinelli M. (2005). Physical-Parameter Design in 2-D MEMS Optical Switches. J. Light. Technol..

[26] Knoernschild C., Kim C., Liu B., Lu F.P., Kim J. (2008). MEMS-based optical beam steering system for quantum information processing in two-dimensional atomic systems. Opt. Lett..

[27] Lian Z., Horak P., Feng X., Xiao L., Frampton K., White N., Tucknott J.A., Rutt H., Payne D.N., Stewart W. (2012). Nanomechanical optical fiber. Opt. Express.

[28] Yang J., Li X., Yang J., Liu J., Su X. (2010). Polarization-independent bidirectional 4 × 4 optical switch in free space. Opt. Laser Technol..

[29] Lütkenhaus N. (1999). Estimates for practical quantum cryptography. Phys. Rev. A.

[30] Hong C.K., Ou Z.Y., Mandel L. (1987). Measurement of subpicosecond time intervals between two photons by interference. Phys. Rev. Lett..

[31] LaPierre R. (2022). Coherent State on a Beam Splitter. Getting Started in Quantum Optics.

[32] Alsmeyer G., Lovric M. (2011). Chebyshev’s Inequality. International Encyclopedia of Statistical Science.

